# The Key Enzyme of the Sialic Acid Metabolism Is Involved in Embryoid Body Formation and Expression of Marker Genes of Germ Layer Formation

**DOI:** 10.3390/ijms141020555

**Published:** 2013-10-14

**Authors:** Wenke Weidemann, Jessica Hering, Dorit Bennmann, Annett Thate, Rüdiger Horstkorte

**Affiliations:** Institut für Physiologische Chemie, Martin-Luther-Universität Halle-Wittenberg, Hollystr.1, Halle D-06114, Germany; E-Mails: wenke.weidemann@medizin.uni-halle.de (W.W.); jessica.hering@web.de (J.H.); dorit.bennmann@medizin.uni-halle.de (D.B.); annett.thate@medizin.uni-halle.de (A.T.)

**Keywords:** sialic acid biosynthesis, UDP-*N*-acetyl-2-epimerase, *N*-acetylmannosamine kinase, embryonic stem cells, embryoid bodies, gene expression

## Abstract

The bi-functional enzyme UDP-*N*-acetyl-2-epimerase/*N*-acetylmannosamine kinase (GNE) is the key enzyme of the sialic acid biosynthesis. Sialic acids are negatively charged nine carbon amino sugars and are found on most glycoproteins and many glycolipids in terminal positions, where they are involved in a variety of biological important molecular interactions. Inactivation of the GNE by homologous recombination results in early embryonic lethality in mice. Here, we report that GNE-deficient embryonic stem cells express less differentiation markers compared to wild-type embryonic stem cells. As a result, GNE-deficient embryonic stem cells fail to form proper embryoid bodies (EB) within the first day of culture. However, when culturing these cells in the presence of sialic acids for three days, also GNE-deficient embryonic stem cells form normal EBs. In contrast, when culturing these cells in sialic acid reduced medium, GNE-deficient embryonic stem cells proliferate faster and form larger EBs without any change in the expression of markers of the germ layers.

## 1. Introduction

Sialic acid (Sia) belongs to a family of nine carbon amino sugars with more than 50 members. Sia is expressed as terminal sugars on glycoproteins on the cell surface and on secreted glycoproteins and involved in many biological interactions [1]. The precursor for all Sia is *N*-acetylneuraminic acid (for review see: [2,3]). *N*-Acetylneuraminic acid is synthesized in the cytosol from UDP-*N*-acetylglucosamine by four sequential reactions. The key reactions are catalyzed by one bi-functional enzyme, the UDP-*N*-acetylglucosamine-2-epimerase/*N*-acetylmannosamine kinase (GNE) [4,5]. GNE is ubiquitously expressed with highest expression in the liver [5–7] and very early during development and in all stages investigated [6]. Homozygous (−/−) GNE inactivation by classical gene targeting causes early embryonic lethality at embryonic day 8.5 [8], whereas heterozygous (+/−) GNE-deficient mice are vital, although the overall sialylation is reduced with the exception of kidney [9].

Several recent studies suggest that GNE itself and the sialic acid metabolism play an important role in cell regulation and gene expression [10–12]. By the use of homozygous GNE-deficient (−/−) embryonic stem cell lines in combination with Sia precursors, it could be demonstrated recently that both GNE and Sia is involved in proliferation, gene expression and cell differentiation, such as neurite outgrowth [11,12]. In addition, using the same embryonic stem cell lines, it could be demonstrated that GNE is involved in early development of skeletal and cardiac muscles [13]. This is of particular interest, since mutations within the GNE gene are responsible for the hereditary inclusion body myopathy or GNE-myopathy, a rare and unique recessive neuromuscular disorder characterized by late onset and progressive muscle weakness [14]. However, the misguided skeletal muscle differentiation in GNE-deficient embryonic stem cells seems not to be the cause of the early lethality of GNE-deficient mice embryos at day E8.5. Mice, which did not develop skeletal muscles, died perinatal [15,16]. Although the GNE-deficient ES cells show a reduced expression of MyoD, this could be compensated by another myogenic transcription factor, Myf5, without any consequence on muscle differentiation or embryogenesis [17]. Therefore we analyzed in this study very early embryonic development and present data that GNE-deficient embryonic stem cells express less differentiation markers, such as the ectoderm marker Nestin (for review: [18]), the mesoderm marker Nodal (for review: [19]), the cardiac marker Nkx2,5 (for review: [20]) and the marker of extra embryonic tissue, cdx2 [21]. Furthermore, we studied early stages of development ES cells in three-dimensional structures, in so-called embryoid bodies (EB), which recapitulate the development of the three germ layers *in vitro* [22]).

When trying to culture GNE-deficient embryonic stem cells in hanging drops [23] it turned out that they formed much smaller embryoid bodies compared to control (=wildtype) embryonic stem cells. However, after culturing embryoid bodies for three days in Sia-containing medium, GNE-deficient embryonic stem cells formed normal embryoid bodies and expression of the differentiation markers was the same compared to control embryonic stem cells. Previously we could demonstrate that GNE-deficient embryonic stem cells proliferate much faster under Sia-free conditions [12]. Therefore, we cultured these cells in hanging drops in defined, Sia-reduced medium. Under these conditions, GNE-deficient embryonic stem cells form larger embryoid bodies and interestingly we found an increase in the expression of the differentiation markers Nkx2,5 and Cdx-2.

## 2. Results and Discussion

### 2.1. Characterization of GNE-Deficient Embryonic Stem Cells

In this study, we used wildtype and GNE-deficient embryonic stem cells generated as sister cell lines from one heterozygous GNE-deficient mouse. These cell lines were characterized before [12] and retested before starting the experiments shown below. As expected, only the wild-type cell line expressed GNE, GNE-mRNA or GNE protein and has GNE-enzyme activity (data not shown), but both cell lines expressed similar m-RNA amounts of the stem markers Oct4, Sox2, the SSEA-1 antigen and alkaline phosphatase activity (data not shown).

### 2.2. GNE-Deficient Embryonic Stem Cells Express Less Differentiation Markers

**Figure 1:**
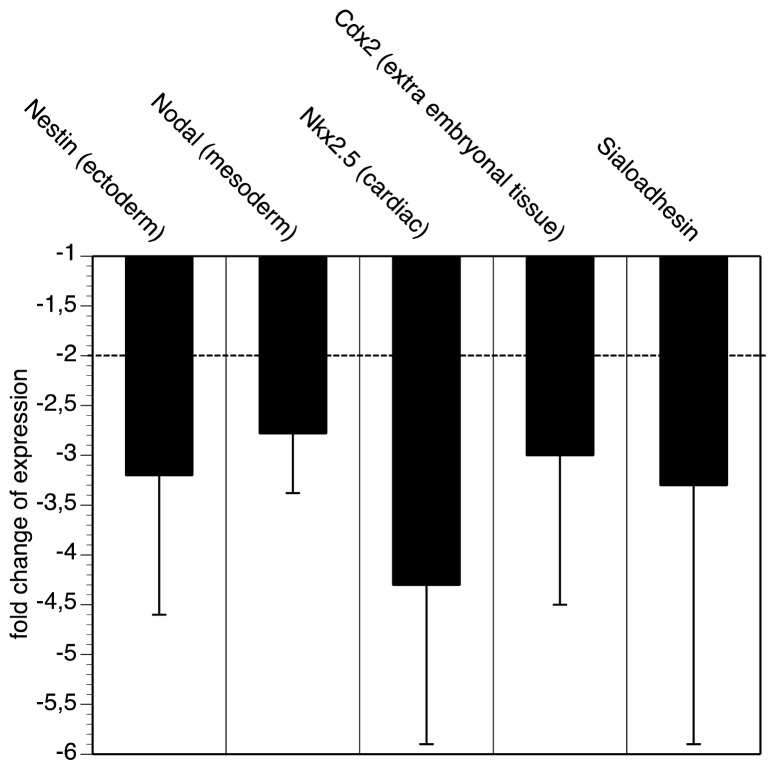
qRT-PCR analysis. Wild-type and GNE-deficient (−/−) embryonic stem cells were cultured in fetal calf serum containing medium and analyzed by qRT-PCR for expression of Nestin, Nodal, Nkx2,5, Cdx-2 and Sialoadhesin. Bars represent fold change of expression of GNE-deficient embryonic stem cells compared to wild-type embryonic stem cells. Each experiment was performed twice in triplicates.

In a first series of experiments we analyzed and compared the expression of marker genes between wildtype and GNE-deficient embryonic stem cells. We have chosen the following differentiation markers: the ectoderm marker Nodal, the mesoderm marker Nestin, the cardiac marker Nkx2,5 and Cdx2 as extra embryonal tissue marker. Although both cell lines express similar levels of stem cell markers (see above), the GNE-deficient embryonic stem cells are much more immature indicated by lower expression of all four selected differentiation (marker) genes (Figure 1). The down-regulation of all genes is between three- and fourfold. Furthermore, we quantified the expression of Sialoadhesion, a C-type sialic acid binding lectin, which is very high expressed in P19 embryonic stem cells [24] and beta-1 integrin [25], a crucial cell adhesion molecule for development. Whereas the expression of the Sialoadhesion transcript is down-regulated (Figure 1), the expression of beta1 integrin is unchanged (not shown). Taken together, we realized that GNE-deficient embryonic stem cells are more immature compared to wild-type embryonic stem cells. This was shown by the reduction of germ layer specific transcripts Nodal, Nestin, Nkx,2,5, or Cdx-2.

### 2.3. Embryoid Body Formation of GNE-Deficient Embryonic Stem Cells Is Retarded

**Figure 2:**
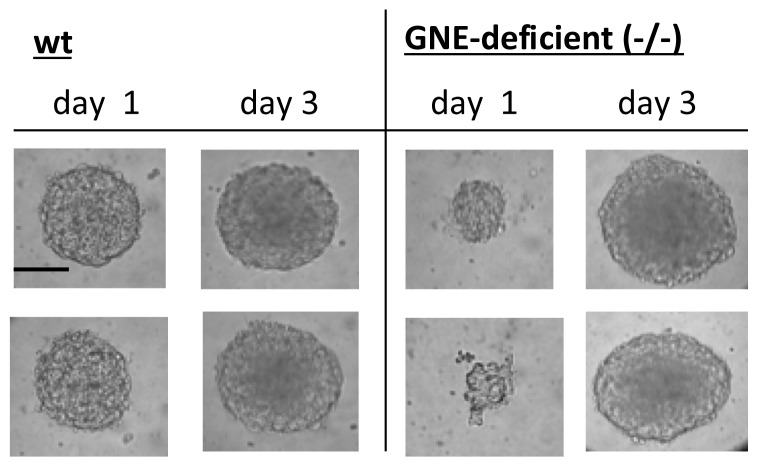
Embryoid body formation. Wild-type (wt) and GNE-deficient embryonic stem cells (GNE-deficient (−/−)) were cultured in hanging drops in FCS containing medium for 24 h or 72 h. Representative micrographs were shown. Bar = 100 μm.

Embryonic stem cells are known to form so-called embryoid bodies after culture in hanging drops. In the next step tested wildtype and GNE-deficient embryonic stem cells to form embryoid bodies. After one day of culture in hanging drops, GNE-deficient embryonic stem cells formed much smaller embryoid bodies compared to wildtype embryonic stem cells. However, after three days of culture both embryonic stem cells lines form similar and undistinguishable embryoid bodies (Figure 2). We then quantified the mRNA expression of the selected differentiation marker genes, Sialoadhesin and beta1 integrin in embryoid bodies of wildtype and GNE-deficient embryoid bodies after three days in hanging drop culture, which were shown in Figure 2. We found no differences in expression of the differentiation marker genes Nestin, Nodal, Nkx2,5, Cdx-2 between wildtype and GNE-deficient embryoid bodies. Also the expression of Sialoadhesion transcript was not different between wildtype and GNE-deficient embryoid bodies (data not shown). However, beta1 integrin expression was 2.2-fold higher in GNE-deficient embryoid bodies compared to wild-type embryoid bodies.

These data imply that cell adhesion molecules, such as integrins are involved in the retardation of embryoid body formation of GNE-deficient embryonic stem cells.

### 2.4. Embryoid Body Formation of GNE-Deficient Embryonic Stem Cells Is Sialic Acid Dependent

**Figure 3:**
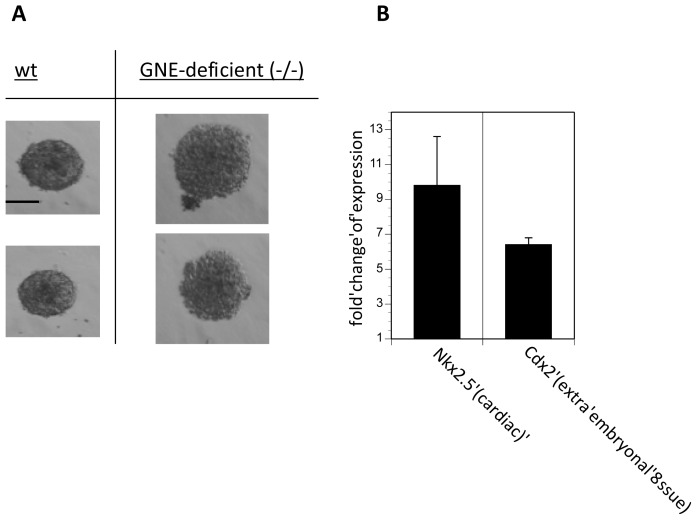
Embryoid body formation and qRT-PCR analysis in the absence of sialic acids. Wild-type (wt) and GNE-deficient (GNE-deficient (−/−)) embryonic stem cells were cultured in hanging drops in sialic acid free serum replacement medium for 72 h. (**A**) Representative micrographs were shown. Bar = 100 μm; (**B**) Bars represent fold change of expression of GNE-deficient embryonic stem cells compared to wild-type embryonic stem cells. Each experiment was performed twice in triplicates.

Recently, we found that GNE-deficient embryonic stem cells proliferate much faster in Sia-free or Sia-reduced culture media. Therefore, we analyzed the embryonic body formation in Sia-reduced SR (=serum replacement) culture medium (note that full FCS-containing culture medium is very enriched in Sia; see: [12]). Whereas wildtype embryonic stem cells do not distinguish between full Sia FCS containing medium and Sia-reduced SR medium, GNE-deficient embryonic stem cells form much larger embryoid bodies after three days of culture in Sia-reduced medium (Figure 3a). When quantifying the expression of the differentiation marker genes, we found an 10-fold up-regulation of Nkx2,5 and an 6-fold up-regulation of Cdx-2 in GNE-deficient embryoid bodies (Figure 3B). Expression of Nodal, Nestin, Sialoadhesion and beta-1 integrin is not different between wildtype or GNE-deficient embryoid bodies (data not shown). In summary, we were able to culture embryoid bodies in serum replacement medium. This medium contains only very low concentration of Sia compared to FCS-containing medium [12]. In the same study [12] we already demonstrated that the Sia content of the medium is involved in proliferation of embryonic stem cells. GNE-deficient embryonic stem cells proliferate much faster compared to wildtype embryonic stem cells in the absence of Sia. In addition, the presence of Sia-precursors interferes with proliferation. In the present study we made a similar observation: embryonic bodies of GNE-deficient embryonic stem cells grow faster in the absence of Sia. Unfortunately, long-term culture (longer than two days) of embryonic bodies in the absence of Sia is not possible because cells undergo apoptosis in complete serum-free medium (data not shown). Our understanding is that embryoid body formation is more comparable to early embryogenesis than to later development processes. In all *in vitro* experiments such as that described here, there is one very important point to mention: the placenta. The placenta is responsible for the supply of the embryo, whereas under all *in vitro* conditions the cells of the embryoid bodies are forced to use all supplement direct from the medium and no cellular mechanism can supplement missing components, such as Sia. Here, it is noteworthy that the placenta expresses high quantities of GNE [6]. Therefore, Sia might be responsible for the proper function of the placenta by itself or Sia synthesized by the placenta are used for generation of highly sialylated proteins provided for the embryo.

## 3. Experimental Section

### 3.1. Cell Cultures

Mouse embryonic stem cells were isolated as described in Schwarzkopf *et al*. [8]. Cells were cultivated on gelatine-coated flasks in DMEM (PAA, Cölbe, Germany) containing 15% FCS (PAA, Cölbe, Germany), 2000 U/mL leukaemia inhibitory factor (Merck Millipore, Darmstadt, Germany), 0.1 mM β-mercaptoethanol (Sigma-Aldrich, Taufkirchen, Germany, 0.2 mM non-essential amino acids (PAA, Cölbe, Germany), 2 mM L-glutamine (PAA, Cölbe, Germany) and nucleosides (PAA, Cölbe, Germany). Cells were grown to 80% confluency and then split 1:2 or 1:3. Cells were passaged every second day and were cultivated in a humidified atmosphere at 37 °C and 5% CO_2_. Because cells are able to metabolize bound sialic acids from serum of the medium, two different cultivation conditions were established. First, the cells were cultured in medium containing 15% FCS, and, second, cells were cultured in serum-free medium (SF), where FCS was replaced by serum replacement (Gibco Life technologies, Darmstadt, Germany) containing only low concentration of sialic acid content compared to FCS.

### 3.2. Embryoid Body Formation

Embryonic stem cells are able to generate Embryoid bodies (EBs) if their differentiation is not inhibited by LIF (Leukemia inhibitory factor). These EBs develop cells of all three germ layers (Ektoderm, Endoderm, Mesoderm) [26]. The hanging-drop method was used to generate EBs [27]. Therefor 1000 embryonic stem cells per 20 μL drop were plated on a cover of a 10 cm bacterial dish filled with PBS. Differentiation medium contains DMEM, 20% FCS or SR, 0.1 mM β-mercaptoethanol, 0.2 mM non-essential amino acids and 2 mM l-glutamine. Nearly 100 drops on each cover were plated. Plating day was defined as day 0. At day 3 after plating EBs were collected and washed in PBS and EBs from three plates were pooled for RNA isolation.

### 3.3. qRT-PCR

Total RNA was extracted from embryonic stem cells and embryoid bodies using TRIzol reagent (Invitrogen, Darmstadt, Germany). Five microgram total RNA were transcribed in cDNA using SuperScriptII reverse transcriptase (Invitrogen, Darmstadt, Germany) according to the manufacturer’s instructions and 5 μL of a 1:5 dilution of the cDNA was used for quantitative real-time PCR (qRT-PCR). PCR reaction was performed with iQ.5 Multicolor Real-Time PCR Detection System (BioRad, München, Germany) using the SuperMix qPCR Green (Jena Bioscience, Jena, Germany) according to the manufacture’s instruction. The initial denaturation step was 95 °C for 3 min following 40 cycles of 95 °C for 10 s, 60 °C for 30 s and 72 °C for 30 s with fluorescence detection after each cycle. Then melting curve was done with a temperature gradient from 60 °C to 95 °C (1 °C per 10 s). The content of selected genes was normalized to the housekeeping gene (*HKG*) *GAPDH*. Calculation was performed using the comparative *C*t method according to:

- Δ*C*t_WT_ = *C*t_selected gene WT_ − *C*t_HKG WT_ and Δ*C*t_KO_ = *C*t_selected gene KO_ − *C*t_HKG KO_
- ΔΔ*C*t = Δ*C*t_KO_ − Δ*C*t_WT_
- Ratio = 2^−ΔΔ^*^C^*^t^ (maximum efficiency is assumed)
- Ratio = 1, no different gene expression in KO compared to WT
- Ratio > 1, increased gene expression in KO compared to WT
- Ratio < 1, decreased gene expression in KO compared to WT; in this case negative reciprocal value (−1/Ratio) was used for better representation.

All PCR products were checked by melting curve analysis and DNA gel electrophoresis to exclude the possibility of multiple products or incorrect product size. PCR analysis was conducted in triplicate for each sample. Two different samples were used for each cell type and cultivation condition. The PCR primers and PCR product size are listed in Table 1 below.

## 4. Conclusions

In conclusion we present evidence that the sialic acid biosynthesis and its key enzyme GNE are involved in the embryoid body formation and differentiation of embryonic stem cells. This was demonstrated by quantification of germ layer marker genes and by culturing wildtype- and GNE-deficient (−/−) embryonic stem cells as embryonic bodies in the presence or absence of sialic acids within the culture media. In summary, we found a sialic acid biosynthesis dependent dis-regulated expression of cardiac and extra-embryonal genes.

## Figures and Tables

**Table 1:** List of all primers used.

Name	Primers	Fragment length
GAPDH fwd.	5′-CCT TCA TTG ACC TCA ACT AC-3′	259 bp
GAPDH rev.	5′-GAG ATG ATG ACC CTT TTG GC-3′	

Beta1-Integrin fwd.	5′-TTC AGA CTT CCG CAT TGG CTT TGG-3′	117 bp
Beta1-Integrin rev.	5′-TGG GCT GGT GCA GTT TTG TTC AC-3′	

Sialoadhesin fwd.	5′-GGT GTT GAG GTG GGA GGA GAG-3′	50 bp
Sialoadhesin rev.	5′-GAT GGA CTA GCA GAA AGG GGT TAT GAA-3′	

Nestin fwd.	5′-GAG AAG ACA GTG AGG CAG ATG AGG TTA-3′	113 bp
Nestin rev.	5′-GCC TCT GTT CTC CAG CTT GCT-3′	

Nodal fwd.	5′-GGA GTT TCA TCC TAC CAA CC-3′	386 bp
Nodal rev.	5′-TCC TGC CAT GCC ACG GTA GC-3′	

Nkx-2,5 fwd.	5′-CGA CGG AAG CCA CGC GTG CT-3′	181 bp
Nkx-2,5 rev.	5′-CCG CTG TCG CTT GCA CTT G-3′	

Cdx-2 fwd.	5′-GCA GTC CCT AGG AAG CCA AGT GA-3′	162 bp
Cdx-2 rev.	5′-CTC TCG GAG AGC CCA AGT GTG-3′	
